# Engaging and Mobilizing Community Members to Prevent Obesity Among Adolescents

**Published:** 2009-06-15

**Authors:** Patricia E. Thompson-Reid

**Affiliations:** Centers for Disease Control and Prevention, National Center for Chronic Disease Prevention and Health Promotion, Division of Diabetes Translation

## Abstract

Community-based public health interventions are designed on the premise that the community is an asset in transforming the health system for health protection. One such intervention is Diabetes Today, a training program for health professionals and lay community leaders that has been successful in building awareness of diabetes as a public health problem. We advocate the use of this program to prevent obesity among adolescents.

## Introduction

Obesity is a public health problem because it is preventable, it places a high burden on society, the burden is unevenly distributed in the population, and the public is concerned. The prevalence of obesity is growing among youth in the United States, placing them at great risk for chronic diseases (eg, coronary heart disease, diabetes, arthritis), with associated increases in social and health care costs. To prevent obesity among adolescents, public health efforts should ensure that the conditions are right for youth to make better choices for achieving a healthy weight. The definition of public health by Heller et al (1) recognizes the importance of communities and the role of theory, evidence, and experience in public health practice. How do we use our science to define and respond to implicit and explicit needs of the community? The public health profession has a good track record of using science to identify the health needs of communities; however, it has been less diligent in incorporating the input of communities. It is our responsibility to provide opportunities to engage, empower, and mobilize community members to participate in a process that informs the development of policies, programs, and interventions for public health problems such as obesity. We describe the principles of a program that explicitly recognizes the value of community engagement and show how these principles may be relevant to adolescent obesity prevention.

## The Diabetes Today Program

In 1993, the Centers for Disease Control and Prevention (CDC) launched a community-based program for people with diabetes. Diabetes Today is a training program for health professionals and lay community leaders with the following modules (2):

- Defining and involving the community.
- Assessing the community.
- Selecting an intended audience.
- Prioritizing goals to prevent diabetes and its complications.
- Planning intervention strategies.
- Evaluating the program.

The program's primary goal is to build health professionals' skills in planning and implementing community-based programs for people with diabetes. The major tenets of this training program are community participation, empowerment, and program development and planning. This is not a typical instructor-led training course; rather, participants interact and learn through self-paced reading before and during the course, which requires case studies, practice exercises, role playing, and sharing information and experiences in group discussions. By the end of the course, participants develop a plan to carry out a community intervention, based on the information provided in their case study. Participants are then expected to return to their communities and implement Diabetes Today with community leaders. This program was designed to expand, at the community level, public health efforts to reduce the risks and complications of diabetes beyond policy and clinical interventions. Its flexible framework could be adapted to fit different populations, build awareness about diabetes, and mobilize communities. Diabetes Today has been implemented among adults of all ages in several communities throughout the United States and its territories, including the Appalachian region, US-associated Pacific and Caribbean islands, and both sides of the Mexico border (2).

Some communities developed interventions to increase levels of physical activity — particularly walking — among people with diabetes and their families, including adolescent members without diabetes, who are also at risk by virtue of their relationship to the person with diabetes. These grassroots efforts illustrated primary and secondary prevention, with the family as the focus of the intervention. Other interventions focused on increasing the availability of nutritious foods in communities and promoting the adoption of behaviors to improve clinical outcomes. Many of these community-specific interventions were being implemented before the science for diabetes prevention was available. We now have scientific evidence that physical activity, even at moderate levels, reduces the risk for chronic diseases such as diabetes and its associated complications, improves well-being, and helps prevent weight gain and obesity among adults (3).

## Adapting Diabetes Today for Obesity Prevention

We can apply the Diabetes Today framework to obesity prevention among youth by examining the context within which we find adolescents. Generally, they are nested in a family unit, a neighborhood, and a community. Public health interventions can occur at many levels, but this framework focuses on the community, and the intended audience is adolescents. Thus, we must consider youth development practice and theory and employ strategies to prevent obesity that provide opportunities, skills, and support in a safe environment.

Health promotion practices aim to empower people so they can gain control over the underlying factors that influence health. Community-based public health interventions have used many health promotion theories and practices, such as empowerment, community participation, social marketing, and community-based participatory research, all based on the premise that the community is an asset in transforming the health system for health protection. The community is also the place where the social determinants of health converge to affect behavior and health. Therefore, the participation of community members is imperative in a process to bring about change in shared outcomes. This participatory process begins with the identification of an issue or concern, continues through the development and evaluation of an intervention, and is a continuous cycle of assessment, planning, action, evaluation, and learning. This process takes time and commitment from both the practitioner and members of the community.

Some communities may be offended by the words "obesity" or "overweight" to describe adolescents because of culture, health beliefs, or community norms. Demonstrating respect by allowing community members to use their own words and share their knowledge and experiences opens opportunities for better communication, trust, creativity, and innovation toward developing appropriate community-specific interventions. For example, the built environment is associated with physical activity (4), and regular physical activity among adolescents leads to better risk profiles for heart disease, diabetes, bone health, and depression (5,6). However, even though parents may be aware that their children should be more physically active, they may be reluctant to allow them to participate in after-school sports because of concerns about the safety of walking home from school (7). If public health practitioners were aware of parental concerns, they could provide opportunities for adolescents and their families to communicate issues related to safety and inactivity to stakeholders, exchange ideas, and begin to build the community's capacity to solve this problem. Ultimately, community members would be more willing to advocate for change and participate in the decision-making process, become more aware of how to access resources for solving problems, and be better able to solve other problems that might arise (8).

Adapting the Diabetes Today framework to obesity prevention among youth requires us to look closely at the physical environment where adolescents live, work, and play. More research on the physical environment's impact on physical activity and nutritional practices in youth is needed. In the meantime, in keeping with the recommendations of the CDC's Guide to Community Preventive Services, we can enhance the environments of adolescents and reduce their risk for injury so they can engage in physical activity in safe environments (7). We can also change social norms so that eating healthy and nutritious foods becomes normative behavior.

Acknowledging that the health sector alone cannot transform any community is important. The Diabetes Today framework emphasizes intersectoral collaboration and partnerships for achieving shared goals. If we use this framework to focus on obesity prevention among adolescents, we must include youth and representatives from organizations that cross several domains involved with youth, such as schools, local grocery stores and fast-food restaurants, religious organizations, and parks and recreation departments. All stakeholders must also accept that youth representatives must be given the opportunity to make appropriate decisions throughout the planning, implementation, and evaluation process.

Generally, adolescents are cared for and influenced by a family, a social network, and their physical environment. In their discussion of social network approaches to preventing childhood obesity in this issue of *Preventing Chronic Disease*, Koehly and colleagues note that "individuals define themselves in terms of their interconnectedness and relationships with their family, friends, neighbors, and community" (9). Understanding these networks and their effect on the behavior of youth in the community is vital when developing interventions that will affect their lives. Public health professionals must also be aware of the rules and regulations associated with working with minors when collaborating with communities to explore possible strategies to prevent obesity (10).

Finally, we must consider the role of parents in interventions involving adolescents, theories about the intergenerational transmission of health risk behaviors from parents to adolescents (11), and ethical arguments implying that parents cause their child's obesity (12). Parents influence their children's well-being, but community-driven interventions or participatory research should not focus on blame as a basis for developing evidence or best practices. Rather, the focus should be on exploring how adult family members and networks influence the transmission of health risk behaviors to adolescents.

## Conclusion

Any model for developing community-based interventions to prevent obesity among adolescents presents a challenge — one that requires systems-based approaches and the involvement of all members of the community, with a focus on youth, their families, and the institutions that serve them. Adapting the Diabetes Today model as a training tool for health professionals could help to assure the availability of public health professionals who can develop a community-based program for preventing and controlling obesity among youth. The ultimate goal is to change community and organizational norms so that healthy behaviors among adolescents become lifelong practices. This could not only reduce chronic diseases in adulthood, but also prepare our youth for leadership and civic responsibility through their participation in decisions that affect their personal health and the health of their communities.
